# Association of Maternal Tobacco Use During Pregnancy With Preadolescent Brain Morphology Among Offspring

**DOI:** 10.1001/jamanetworkopen.2022.24701

**Published:** 2022-08-01

**Authors:** Runyu Zou, Olga D. Boer, Janine F. Felix, Ryan L. Muetzel, Ingmar H. A. Franken, Charlotte A. M. Cecil, Hanan El Marroun

**Affiliations:** 1 Department of Child and Adolescent Psychiatry/Psychology, Erasmus MC, University Medical Center Rotterdam, Rotterdam, the Netherlands; 2 Julius Center for Health Sciences and Primary Care, University Medical Center Utrecht, Utrecht University, Utrecht, the Netherlands; 3 Institute for Risk Assessment Sciences, Utrecht University, Utrecht, the Netherlands; 4 Department of Psychology, Education and Child Studies, Erasmus School of Social and Behavioral Sciences, Erasmus University Rotterdam, Rotterdam, the Netherlands; 5 The Generation R Study Group, Erasmus MC, University Medical Center Rotterdam, Rotterdam, the Netherlands; 6 Department of Pediatrics, Erasmus MC, University Medical Center Rotterdam, Rotterdam, the Netherlands; 7 Department of Epidemiology, Erasmus MC, University Medical Center Rotterdam, Rotterdam, the Netherlands; 8 Molecular Epidemiology, Department of Biomedical Data Sciences, Leiden University Medical Center, Leiden, the Netherlands

## Abstract

**Importance:**

Maternal tobacco use during pregnancy has been associated with various health consequences, including suboptimal neurodevelopment in offspring. However, the effect of prenatal exposure to maternal smoking on child brain development has yet to be elucidated.

**Objective:**

To investigate the association between maternal smoking during pregnancy and offspring brain development in preadolescence as well as the mediating pathways.

**Design, Setting, and Participants:**

This prospective, population-based cohort study was embedded in the Generation R Study, Rotterdam, the Netherlands. The Generation R Study was launched in 2002, with follow-up ongoing. Child brain morphology was assessed at 9 to 11 years of age (ie, 10-12 years between exposure and outcome assessment). Data analysis was performed from March 1, 2021, to February 28, 2022, and at the time of manuscript revision. Participants included the singleton children of pregnant women residing in the study area with an expected date of delivery between April 1, 2002, and January 31, 2006; 2704 children with information on maternal smoking during pregnancy and structural neuroimaging at 9 to 11 years of age were included. A subsample of 784 children with data on DNA methylation at birth was examined in the mediation analysis.

**Exposures:**

Information on maternal smoking during pregnancy was collected via a questionnaire in each trimester. As a contrast, paternal smoking was assessed at recruitment.

**Main Outcomes and Measures:**

Brain morphology, including brain volumes and surface-based cortical measures (thickness, surface area, and gyrification), was assessed with magnetic resonance imaging. For mediation analysis, DNA methylation at birth was quantified by a weighted methylation risk score.

**Results:**

The 2704 participating children (1370 [50.7%] girls and 1334 [49.3%] boys) underwent brain imaging assessment at a mean (SD) age of 10.1 (0.6) years. Compared with nonexposed children (n = 2102), exposure to continued maternal smoking during pregnancy (n = 364) was associated with smaller total brain volume (volumetric difference [*b*] = −14.5 [95% CI, −25.1 to −4.0] cm^3^), cerebral gray matter volume (*b* = −7.8 [95% CI, −13.4 to −2.3] cm^3^), cerebral white matter volume (*b* = −5.9 [95% CI, −10.7 to −1.0] cm^3^), and surface area and less gyrification. These associations were not explained by paternal smoking nor mediated by smoking-associated DNA methylation patterns at birth. Children exposed to maternal smoking only in the first trimester (n = 238) showed no differences in brain morphology compared with nonexposed children.

**Conclusions and Relevance:**

The findings of this cohort study suggest that continued maternal tobacco use during pregnancy was associated with lower brain volumes and suboptimal cortical traits of offspring in preadolescence, which seemed to be independent of shared family factors. Tobacco cessation before pregnancy, or as soon as pregnancy is known, should be recommended to women for optimal brain development of their offspring.

## Key Points

**Question:**

Is maternal tobacco use during pregnancy associated with preadolescent brain morphology among offspring?

**Findings:**

In this cohort study of 2704 children in the Netherlands, exposure to continued maternal smoking during pregnancy was associated with lower global and regional brain volumes as well as smaller surface area and less gyrification at 9 to 11 years of age.

**Meaning:**

These findings suggest that maternal smoking throughout pregnancy may be associated with suboptimal brain development among offspring in the long term.

## Teaser

This cohort study investigates the association between maternal tobacco use during pregnancy and brain morphology, including volumetric measures and cortical metrics, in offspring during preadolescence.

## Introduction

Maternal tobacco use during pregnancy affects the health of not only the mother but also her offspring. Despite the well-documented child growth consequences,^1^ accumulating evidence suggests that maternal tobacco use during pregnancy is also associated with suboptimal neurodevelopment of offspring, including impaired cognitive abilities, bipolar disorder, and schizophrenia spectrum disorders, but shared genetic and family factors hamper causal inference.^2,3,4^

Differences in structural brain development may underlie the observed neurocognitive outcomes. A prospective study including 232 preterm infants^5^ found associations of prenatal maternal smoking with smaller frontal lobe and cerebellum at corrected term age. Using a retrospective design,^6^ a smaller amygdala was observed in 180 adolescents prenatally exposed to maternal smoking compared with 198 without exposure, although no difference was found in total brain volume (TBV). In a subgroup of our own cohort in an earlier phase,^7^ maternal smoking throughout pregnancy was associated with less TBV and cortical gray matter volume, as well as thinner cortices (primarily in the frontal and parietal lobes) in 97 exposed children and 113 nonexposed controls aged 6 to 8 years. However, the small sample sizes of the aforementioned research made it difficult to capture subtle effect sizes, and investigations in specific groups (eg, preterm children) limit the generalizability of the findings. In addition, more efforts should be made to investigate the causal nature of observed relations, given the confounding issues in research on maternal smoking during pregnancy and child outcomes.^8^ Using paternal smoking as a negative control is a well-known approach to examine whether there is a direct intrauterine effect of a maternal exposure such as smoking. Similar maternal and paternal effects suggest that there may be familial or genetic confounding factors, whereas a larger maternal vs paternal effect supports a direct intrauterine effect.^9^

Moreover, biological mechanisms underlying potential effects of tobacco exposure at the molecular level remain unclear. Interestingly, tobacco exposure is arguably one of the most significant environmental factors associated with DNA methylation alterations in both neonates (prenatal exposure) and adults (direct smoking).^10^ For example, a genome-wide consortium meta-analysis including 13 cohorts^11^ found that persistent maternal smoking during pregnancy was associated with more than 6000 differentially methylated 5′-C-phosphate-G-3′ (CpG) sites in neonates, many of which were mapped to genes involved in neural development. However, whether DNA methylation explains the observed associations between maternal smoking during pregnancy and offspring brain development remains unknown.

The present study aimed to investigate the prospective association between maternal tobacco use during pregnancy and brain morphology, including volumetric measures and cortical metrics in preadolescents. Paternal smoking during pregnancy was used as a negative control to test for shared genetic and family confounding. We also explored whether previously identified smoking-associated DNA methylation patterns at birth mediate any observed associations.

## Methods

### Setting and Participants

This cohort study was embedded in the Generation R Study, a prospective population-based study in Rotterdam, the Netherlands. Pregnant women with an expected delivery date between April 1, 2002, and January 31, 2006, residing in the study area were eligible for recruitment.^12^ The Generation R Study is approved by the Medical Ethical Committee of Erasmus MC, University Medical Center Rotterdam. Written informed consent was obtained from all participants. This study followed the Strengthening the Reporting of Observational Studies in Epidemiology (STROBE) reporting guideline.

The Generation R Study recruited a total of 9778 women who gave birth to 9506 live singletons. Among these children, 2704 with information on maternal tobacco use during pregnancy and usable brain magnetic resonance imaging (MRI) data at 9 to 11 years of age were included in the study population. eFigure 1 in the Supplement depicts the study population selection.

### Maternal and Paternal Tobacco Use

Postal questionnaires were used to collect information on maternal tobacco use in each trimester.^13^ Maternal tobacco use during pregnancy was categorized based on all 3 questionnaires as (1) never during pregnancy, (2) until pregnancy was known, and (3) continued during pregnancy. For mothers enrolled during the postnatal period (n = 201), information on tobacco use during pregnancy was retrospectively obtained (with the same categorization). We also collected information on the frequency of tobacco use for the smoking mothers.

Information on paternal tobacco use preceding the pregnancy was obtained from both the mother and the partner using questionnaires at enrollment. Maternal- and self-reported paternal smoking status showed high agreement: 1606 of 1721 pairs (93.3%) provided the same answer.

### Neuroimaging

Before the MRI scan, children underwent a mock scanning procedure to become familiar with the neuroimaging assessment. Brain images were acquired using the same sequence on the same 3.0-T MRI scanner (Discovery MR750; GE Healthcare) using an 8-channel head coil. Following a 3-plane localizer scan, T1-weighted structural images were acquired with an inversion recovery–prepared fast spoiled gradient recalled sequence. Further information on the sequence and neuroimaging procedure is described elsewhere.^14^

Volumetric segmentation and cortical reconstruction were performed using the FreeSurfer software suite.^15^ The standard reconstruction stream was applied, and surface-based models of white matter and gray matter were generated. The quality of surface reconstruction was visually inspected, after which data with insufficient quality were eliminated.^16^

### Covariates

Based on previous studies,^6,7,17^ we included the following covariates: child sex and age at neuroimaging, maternal age at enrollment, ethnicity (categorized as Dutch, non-Dutch western, and non-Dutch nonwestern according to the classification of Statistics Netherlands), educational level (categorized into primary, secondary, and higher education), marital status, parity, prepregnancy body mass index, psychopathology score, alcohol use, and household net monthly income. The psychopathology score was based on the Global Severity Index derived from the Brief Symptom Inventory (range, 0-4, with higher scores indicating more clinically relevant psychological symptoms).^18^ Child sex and date of birth were obtained from medical records. Maternal prepregnancy body mass index was calculated by self-reported prepregnancy weight and height measured at enrollment. Information on all other covariates was collected with questionnaires during pregnancy.

### Methylation Risk Score

Variance in DNA methylation was indexed using a methylation risk score (MRS). In brief, DNA was extracted from cord blood, and samples were processed with the Infinium HumanMethylation450 BeadChip array (Illumina Inc) followed by standardized laboratory quality control.^19^ We quantified DNA methylation at each CpG site. Among the 6073 CpG sites that were associated with continued maternal smoking during pregnancy with false discovery rate significance,^11^ 5643 were available in 784 participants of European ancestry in our sample and were included to construct the weighted MRS. We also constructed an MRS using 551 CpG sites that survived a more stringent Bonferroni correction to compare results. In addition, we constructed a second MRS consisting specifically of CpG sites (405 with false discovery rate significance and 62 with Bonferroni significance) that in addition to showing associations with maternal smoking during pregnancy were also annotated to genes listed as part of the brain development pathway (GO:0007420). Information on plate number to indicate batch, cell types, and 4 genetic principal components of ancestry was collected as additional covariates in analyses of MRS.^20^

### Statistical Analysis

Data were analyzed from March 1, 2021, to February 28, 2022. For descriptive purposes, continuous variables are presented as mean (SD) and categorical variables as No. (%). In the nonresponse analysis, we compared maternal and child variables between respondents (n = 2704) and nonrespondents (n = 5318) using an unpaired *t* test or Wilcoxon test for continuous variables and a χ^2^ test for categorical variables.

In primary analyses, we used linear regression to examine maternal smoking in early pregnancy only or continued maternal smoking during pregnancy in association with offspring brain morphology at 9 to 11 years of age, with nonexposed children as the reference group. For volumetric measures, we used a hierarchical approach by first examining TBV as a global measure, followed by regional brain volumes including cerebral gray matter and white matter and the cerebellum. Volumetric differences were reported as *b* values. In secondary analyses, we examined subcortical brain volumes including the thalamus, amygdala, hippocampus, putamen, pallidum, caudate, and nucleus accumbens. These subcortical volumes were standardized before analysis owing to the variance in absolute volumes. For surface-based cortical brain measures, we used vertex-wise linear regression with a custom in-house QDECR R package at each cortical vertex to examine cortical thickness, surface area, and gyrification in children.^16,21^ To investigate possible dose-response associations, we further evaluated maternal smoking frequency as a continuum to both volumetric and cortical measures in children born to continued smokers, because use of categorical variables can be subject to unbalanced sample sizes and is less sensitive to detect small effects.^22,23^

In addition, we evaluated paternal smoking preceding pregnancy with child brain outcomes to explore whether any associations were explained by shared genetic or environmental factors. We also conducted a mediation analysis to examine whether the smoking-associated DNA MRS at birth mediates the association between prenatal exposure to maternal smoking and brain morphology at 9 to 11 years of age.

Two sensitivity analyses were performed to assess our findings. First, we used inverse probability weighting to count for potential attrition. Second, we excluded participants included after delivery to rule out recall bias on maternal smoking information.

We used multistage covariates adjustment in the above analyses. Child sex and age at neuroimaging were adjusted for in the minimally adjusted model, and maternal age at enrollment, ethnicity, marital status, educational level, psychopathology score, alcohol use during pregnancy, and household income were additionally adjusted for in the fully adjusted model based on the 5% change-in-estimate criterion.^24^ For subcortical structures, intracranial volume (ICV) was additionally adjusted for in a third model to examine whether any volumetric differences were independent of the global brain size. For mediation analyses using the MRS, we adjusted for child sex and age at neuroimaging, maternal age at enrollment, maternal educational level, cell types, plate number, and genetic principal components.

Missing data on covariates were estimated using multivariate imputation by chained equations,^25^ and we only report pooled results (results from the first imputed data set are reported for the mediation analyses). Statistical significance was set as α < .05 (2-sided), and a false discovery rate correction was applied in the primary analyses to minimize false-positive findings.^26^ For surface-based analysis, correction for multiple testing was performed using built-in Gaussian Monte Carlo simulations.^27^ All statistical analyses were performed using R, version 3.6.2 (R Project for Statistical Computing). In addition to standard packages for general analyses, we used the mediation R package to perform mediation analysis,^28^ which estimates direct and indirect effects and their CIs based on bootstrapped simulations. More information on tobacco use assessment, neuroimaging, and statistical analysis can be found in eMethods in the Supplement.

## Results

### Descriptive Information

Table 1 presents the demographic information of the participants. The 2704 participating children underwent brain MRI assessment at a mean (SD) age of 10.1 (0.6) years and included slightly more girls (1370 [50.7%]) than boys (1334 [49.3%]). Most of the mothers (2102 [77.7%]) never smoked during pregnancy, followed by 364 (13.5%) who continued smoking throughout pregnancy. In addition, 238 women (8.8%) stopped smoking when they were aware of the pregnancy, mostly in the first trimester (ie, before gestational age of 13 weeks). Results of the nonresponse analysis are summarized in eTable 1 in the Supplement.

**Table 1.** Descriptive Information of the Study Population^a^

Characteristic	Study group			
	Never smoked during pregnancy (n = 2102)	Smoked until pregnancy was known (n = 238)	Continued smoking during pregnancy (n = 364)^b^	Overall (N = 2704)
Maternal				
Age at enrollment, mean (SD), y	31.3 (4.7)	30.9 (4.6)	29.6 (5.7)	31.1 (4.9)
Ethnicity				
Dutch	1259 (59.9)	144 (60.5)	184 (50.5)	1587 (58.7)
Non-Dutch western	173 (8.2)	29 (12.2)	31 (8.5)	233 (8.6)
Non-Dutch nonwestern	670 (31.9)	65 (27.3)	149 (40.9)	884 (32.7)
Marital status (with partner)	1931 (91.9)	206 (86.5)	270 (74.2)	2407 (89.0)
Prepregnancy BMI, mean (SD)	23.4 (4.0)	22.9 (3.5)	23.9 (4.6)	23.4 (4.1)
Parity (multipara)	878 (41.8)	66 (27.7)	146 (40.1)	1090 (40.3)
Psychopathology score, mean (SD)^c^	0.2 (0.3)	0.3 (0.3)	0.4 (0.5)	0.3 (0.4)
Educational level				
Primary or below	124 (5.9)	11 (4.6)	39 (10.7)	174 (6.4)
Secondary	782 (37.2)	102 (42.9)	230 (63.2)	1114 (41.2)
Higher	1196 (56.9)	125 (52.5)	95 (26.1)	1416 (52.4)
Alcohol use during pregnancy				
Never	939 (44.7)	40 (16.8)	126 (34.6)	1105 (40.9)
Before pregnancy was known	278 (13.2)	68 (28.6)	46 (12.6)	392 (14.5)
Occasionally	713 (33.9)	92 (38.7)	144 (39.6)	949 (35.1)
Frequently^d^	172 (8.2)	38 (16.0)	48 (13.2)	258 (9.5)
Household net income, €/mo				
<1200	289 (13.7)	24 (10.1)	107 (29.4)	420 (15.5)
1201-2000	311 (14.8)	41 (17.2)	83 (22.8)	435 (16.1)
>2000	1502 (71.5)	173 (72.7)	174 (47.8)	1849 (68.4)
Child				
Age at neuroimaging, mean (SD), y	10.1 (0.6)	10.1 (0.6)	10.2 (0.6)	10.1 (0.6)
Sex				
Male	1032 (49.1)	112 (47.1)	190 (52.2)	1334 (49.3)
Female	1070 (50.9)	126 (52.9)	174 (47.8)	1370 (50.7)

Abbreviation: BMI, body mass index (calculated as weight in kilograms divided by height in meters squared).

^a^ Statistics of the first imputed data set are reported. Unless indicated otherwise, data are expressed as No. (%) of participants. Percentages have been rounded and may not total 100.

^b^ Frequency of smoking included less than 1 cigarette/d (n = 53), 1 to 2 cigarettes/d (n = 43), 3 to 4 cigarettes/d (n = 74), 5 to 9 cigarettes/d (n = 115), 10 to 19 cigarettes/d (n = 69), and 20 or more cigarettes/d (n = 10).

^c^ Scores range from 0 to 4, with higher scores indicating more clinically relevant psychological symptoms.

^d^ Defined as 1 or more glasses of alcohol per week in at least 2 trimesters.

### Maternal Smoking and Child Brain Morphology

Focusing on the fully adjusted models, continued maternal smoking during pregnancy was associated with lower TBV (*b* = −14.5 [95% CI −25.1 to −4.0] cm^3^) in children at 10 years of age, whereas exposure to smoking in early pregnancy only was not associated with TBV (*b* = 0.2 [95% CI, −12.0 to 12.5] cm^3^). Table 2 demonstrates the associations between maternal smoking during pregnancy and regional brain volumes of the child. Children born to mothers who continued smoking during pregnancy also showed smaller cerebral gray matter volume (*b* = −7.8 [95% CI, −13.4 to −2.3] cm^3^) and white matter volume (*b* = −5.9 [95% CI, −10.7 to −1.0] cm^3^) compared with nonexposed children, although no differences were found in cerebellar volume (*b* = −0.8 [95% CI, −2.2 to 0.6] cm^3^). Compared with nonexposed children, those born to women who quit smoking in early pregnancy showed no volumetric differences in these brain regions.

**Table 2.** Associations of Maternal Smoking During Pregnancy With Regional Brain Volumes in Children 10 Years of Age^a^

Maternal smoking during pregnancy	Cerebral gray matter volume		Cerebral white matter volume		Cerebellar volume	
	*b* (95% CI)	*P* value	*b* (95% CI)	*P* value	*b* (95% CI)	*P* value
Minimally adjusted model^b^						
Never	[Reference]	NA	[Reference]	NA	[Reference]	NA
Until pregnancy was known	1.8 (−4.9 to 8.4)	.60	−0.6 (−6.2 to 5.1)	.84	0.6 (−1.0 to 2.2)	.48
Continued	−15.2 (−20.7 to −9.7)	<.001	−10.3 (−15.0 to −5.6)	<.001	−2.3 (−3.6 to −1.0)	<.001
Fully adjusted model^c^						
Never	[Reference]	NA	[Reference]	NA	[Reference]	NA
Until pregnancy was known	0.7 (−5.8 to 7.2)	.83	−1.1 (−6.8 to 4.6)	.70	0.6 (−1.0 to 2.2)	.45
Continued	−7.8 (−13.4 to −2.3)	.006^d^	−5.9 (−10.7 to −1.0)	.02^d^	−0.8 (−2.2 to 0.6)	.26

Abbreviation: NA, not applicable.

^a^ Linear regression was used. The *b* values represent volumetric differences (in cm^3^) of the group that smoked until pregnancy was known (n = 238) or the group that continued smoking (n = 364) compared with the never smoked (reference) group (n = 2102).

^b^ Adjusted for child sex and age at brain assessment.

^c^ Adjusted for child sex and age at brain assessment and maternal ethnicity, age at enrollment, marital status, educational level, psychopathology score, alcohol use during pregnancy, and household income.

^d^ Indicates *P* values that survived a false discovery rate correction for multiple comparisons.

**Figure 1.**
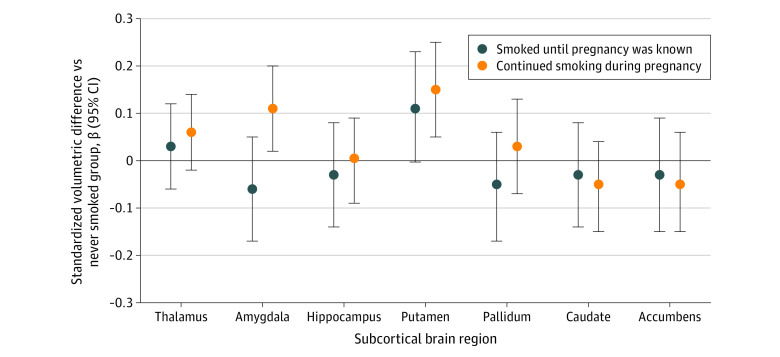
Associations of Maternal Smoking During Pregnancy With Subcortical Brain Volumes in Children 10 Years of Age Differences in standardized subcortical volumes in children aged 10 years who were exposed to maternal smoking compared with nonexposed children. Linear regression models were adjusted for child sex and age at brain assessment, and maternal ethnicity, age at enrollment, marital status, educational level, psychopathology score, alcohol use during pregnancy, household income, and intracranial volume.

Next, we examined the volumes of subcortical structures with and without adjustment for ICV in the regression model. Figure 1 presents the results with adjustment for ICV, showing that maternal smoking throughout pregnancy was associated with larger amygdala and putamen among the children. Interestingly, such differences were not observed without adjustment for child ICV in the amygdala (β = 0.01 [95% CI, −0.1 to 0.1]) or putamen (β = 0.1 [95% CI, −0.03 to 0.2]), suggesting that these 2 structures were relatively larger in children exposed to continued maternal smoking during pregnancy. On the contrary, a smaller caudate (β = −0.2 [95% CI, −0.3 to −0.04]) and nucleus accumbens (β = −0.1 [95% CI, −0.2 to −0.02]) were found among children exposed to continued maternal smoking during pregnancy when ICV was not adjusted for, suggesting differences dependent on global brain size. Among the 364 children exposed to continued maternal smoking during pregnancy, we found no association between maternal smoking frequency during pregnancy and offspring global or regional brain volumes nor volumes of the subcortical structures, suggesting no clear dose-response association.

**Figure 2.**
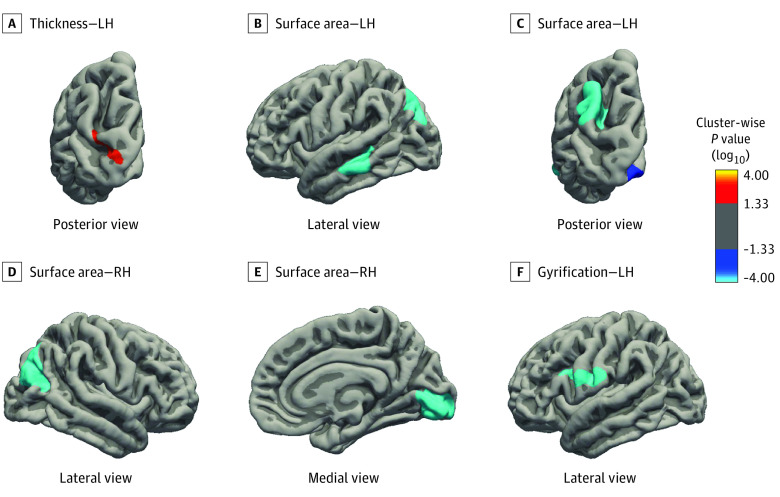
Association of Maternal Smoking During Pregnancy With Cortical Morphology in Children 10 Years of Age Cortical morphology (ie, thickness, surface area, and gyrification) in children aged 10 years born to mothers who continued smoked during pregnancy (n = 364) compared with those born to mothers who never smoked during pregnancy (reference category [n = 2091], excluding children who did not have adequate vertex-wise data). Vertex-wise linear regression was used; the presented model was adjusted for child sex and age at brain assessment and maternal ethnicity, age at enrollment, marital status, educational level, psychopathology score, alcohol use during pregnancy, and household income. Red to yellow brain regions represent larger surface areas, thicker cortices, or more gyrification; dark to light blue regions represent smaller surface areas, thinner cortices, or less gyrification. The colored clusters in this figure all survived a clusterwise (Monte Carlo simulation with 5000 iterations) correction for multiple comparisons (*P* < .001). Statistical details of the associated clusters are provided in eTable 2 in the Supplement. LH indicates left hemisphere; RH, right hemisphere.

The surface-based analysis showed that, compared with nonexposed children, those exposed to continued maternal smoking during pregnancy had thicker cortices in the inferior parietal region of the left hemisphere (*b* = 0.05; 301.6 mm^2^; clusterwise *P* < .001) but smaller surface area in the temporal (*b* = −0.02; 987.1 mm^2^; clusterwise *P* < .001) and occipital (*b* = −0.03; 313.8 mm^2^; clusterwise *P* = .02) lobes of the left hemisphere and the pericalcarine cortex (*b* = −0.04; 1633.5 mm^2^; clusterwise *P* < .001) of the right hemisphere, inferior parietal regions of the left (*b* = −0.03; 1081.9 mm^2^; clusterwise *P* < .001) and right (*b* = −0.04; 1674.3 mm^2^; clusterwise *P* < .001) hemispheres, and less gyrification in the postcentral region (*b* = −0.08; 1017.2 mm^2^; clusterwise *P* < .001) of the left hemisphere (Figure 2 and eTable 2 in the Supplement). Again, we observed no association of maternal smoking in early pregnancy only with cortical thickness, surface area, and gyrification of the child, nor was smoking frequency associated with these cortical metrics.

### Paternal Smoking and Child Brain Morphology

As shown in Table 3, both maternal-reported and self-reported tobacco use of the partner during the periconceptional period were associated with a larger putamen of the child at 10 years of age (also when adjusted for ICV) (*b* = 0.1 [95% CI, 0.04-0.2] cm^3^ and *b* = 0.1 [95% CI, 0.03-0.2] cm^3^, respectively). In addition, maternal-reported paternal smoking was associated with child pallidum (*b* = 0.1 [95% CI, 0.01-0.1] cm^3^), whereas this association was not observed when using paternal self-reported data. Paternal smoking was not associated with other global, regional, or subcortical volumes. Surface-based analyses showed no association of paternal smoking with child cortical thickness, surface area, or gyrification.

**Table 3.** Association of Periconceptional Paternal Smoking With Brain Volumes in Children 10 Years of Age^a^

Brain structure	Maternal-reported paternal smoking			Self-reported paternal smoking		
	No. exposed/unexposed	*b* (95% CI)	*P* value	No. exposed/unexposed	*b* (95% CI)	*P* value
Total brain	1021/1414	−1.4 (−8.7 to 5.9)	.71	728/1028	−4.2 (−12.9 to 4.6)	.35
Cerebral gray matter	1021/1414	−1.3 (−5.2 to 2.6)	.51	728/1028	−3.5 (−8.2 to 1.1)	.13
Cerebral white matter	1021/1414	0.02 (−3.4 to 3.4)	.99	728/1028	−0.2 (−4.2 to 3.9)	.94
Cerebellum	1021/1414	−0.2 (−1.1 to 0.8)	.69	728/1028	−0.5 (−1.6 to 0.6)	.39
Thalamus^b^	1021/1414	−0.01 (−0.1 to 0.04)	.66	728/1028	−0.04 (−0.1 to 0.02)	.19
Amygdala^b^	1021/1414	0.1 (−0.01 to 0.1)	.08	728/1028	−0.01 (−0.1 to 0.1)	.81
Hippocampus^b^	1021/1414	0.03 (−0.04 to 0.09)	.37	728/1028	−0.02 (−0.1 to 0.06)	.62
Putamen^b^	1021/1414	0.1 (0.04 to 0.2)	.002	728/1028	0.1 (0.03 to 0.2)	.01
Pallidum^b^	1021/1414	0.1 (0.01 to 0.1)	.03	728/1028	0.1 (−0.01 to 0.1)	.10
Caudate^b^	1021/1414	0.02 (−0.1 to 0.1)	.66	728/1028	−0.01 (−0.1 to 0.1)	.76
Accumbens^b^	1021/1414	−0.04 (−0.1 to 0.03)	.25	728/1028	−0.1 (−0.2 to 0.01)	.07

^a^ The *b* values represent volumetric differences (in cm^3^) between children exposed to paternal tobacco use vs nonexposed children (ie, the reference group). Standardized differences were used for subcortical structures including the thalamus, amygdala, hippocampus, putamen, pallidum, caudate, and accumbens. Linear regression models were adjusted for child sex and age at brain assessment, and maternal ethnicity, age at enrollment, marital status, educational level, psychopathology score, alcohol use during pregnancy, and household income.

^b^ Additionally adjusted for intracranial volume.

### Mediation Analysis on DNA Methylation

The demographic information of children included in the mediation analysis on DNA methylation is displayed in eTable 3 in the Supplement. Although continued maternal smoking during pregnancy was associated with neonatal MRS, we found no evidence suggesting that neonatal MRS mediated the association between continued maternal smoking during pregnancy and child brain morphology at 10 years of age, using MRS calculated from all CpG sites or CpG sites annotated to genes of the brain development pathway (see eFigure 2 in the Supplement). Consistent results were obtained when using MRS calculated from CpG sites with Bonferroni significance.

### Sensitivity Analysis

Analyses using inverse probability weighting yielded consistent results: compared with children without exposure, those exposed to continued maternal smoking during pregnancy showed a smaller TBV (*b* = −15.7 [95% CI, −26.2 to −5.1] cm^3^) and lower volumes of cerebral gray and white matter after full adjustment for covariates (eTable 4 in the Supplement). Similarly, analyses in children born to women included during pregnancy only (n = 2503) showed smaller TBV (*b* = −13.8 [95% CI, −24.6 to −3.1] cm^3^), smaller volumes of cerebral gray matter (*b* = −7.4 [95% CI, −13.1 to −1.7] cm^3^) and white matter (*b* = −5.5 [95% CI, −10.5 to −0.6] cm^3^) (eTable 5 in the Supplement), and smaller surface area and less gyrification (eTable 2 and eFigure 3 in the Supplement) in children born to women who continued smoking during pregnancy than those born to women who never smoked during pregnancy, suggesting that our findings were not subject to recall bias.

## Discussion

In this study, continued maternal tobacco use during pregnancy was associated with smaller global and regional brain volumes as well as smaller cortical surface area and less gyrification in 10-year-old children. Importantly, we found no evidence suggesting that DNA methylation at birth, when indexed by a prenatal smoking–associated MRS, mediated these associations. No associations of exposure to early-pregnancy smoking with offspring brain morphology were observed. Furthermore, no evident association between paternal tobacco use and child brain morphology was observed. These findings suggest that persistent tobacco exposure in utero could compromise brain development 10 years later, which is unlikely to be explained by shared genetics or family factors.

Previous studies have shown that exposure to continued maternal tobacco use during pregnancy is associated with reduced growth of the head and smaller cerebellar size in fetal life^13^ as well as smaller TBV, smaller cortical gray matter volume, and thinner cortices in early childhood (ie, age 6-8 years).^7^ In the present study, we showed that the reduction in global and regional brain volumes in children exposed to continued maternal smoking during pregnancy remained at 9 to 11 years of age. This is in line with the findings from Rivkin et al^17^ suggesting lower total parenchymal volume and cortical gray matter volume in children aged 10 to 14 years exposed to maternal cigarette use in utero. Interestingly, in contrast to previously cited studies^7,13^ and a study by Toro et al^29^ reporting thinner cortices in children aged 6 to 8 years and 15-year-old adolescents, the most notable cortical findings were smaller surface areas and less gyrification in the present study sample. The nonlinear pattern of cortical development, characterized by initial thickening in childhood and accelerated thinning in adolescence, may account for the heterogeneous findings in cortical thickness and hamper straightforward interpretations.^30,31^ Importantly, in addition to adjusting for various sociodemographic and lifestyle factors, we used a negative control (ie, paternal smoking) in a separate analysis to address confounding bias, which is uncommon in neuroimaging studies.

Consistent with previous studies^7,13^ showing no differences in fetal head growth or brain morphology at 6 to 8 years of age, we did not observe any brain morphological differences in children exposed to smoking in early pregnancy only. Therefore, we speculate that exposure to tobacco throughout pregnancy is more relevant for brain development than transient exposure in early gestation. This can be explained by the fact that human brain development in the embryonic period (ie, until 8 weeks of gestation) is primarily characterized by the differentiation of the neural progenitors and formation of the neural tube, thus exposure to maternal smoking at this stage exerts less impact on brain expansion than later in gestation, when substantial neurogenesis takes place.^32^ In practice, this finding suggests that smoking cessation as soon as pregnancy is known, if not before pregnancy, is not too late for offspring brain development, which is an important public health message for pregnant smokers and their health care practitioners, such as midwives and obstetricians.

Several mechanisms could underlie the associations of intrauterine exposure to smoking with brain development at 10 years of age. First, nicotine is the major psychoactive compound and is known to interfere with neurodevelopment as a neuroteratogen.^33^ Animal models have shown that prenatal nicotine exposure induces apoptotic cell death and decreases cell size in various brain regions,^34,35^ which may lead to a smaller brain of the fetus that persists in the long term. Second, a recent study^36^ suggested an adverse effect of tobacco exposure on bone growth in early life, which may cause a reduced skull capacity that originates in fetal life and persists in childhood and thus restricts brain development in the long term. Last, the prenatal period is a time of considerable epigenomic plasticity that is relevant for human brain development both in utero and after birth.^37,38^ In the present study, neonatal DNA methylation patterns known to associate with prenatal maternal smoking, here indexed by the MRS, did not mediate the association between prenatal exposure to maternal smoking and brain morphology at 10 years of age. However, this potential mechanism cannot yet be excluded, because we did not systematically examine smoking-related CpG sites at an individual level, some of which may play a more profound role in neurodevelopment.^39^ In addition, research has found maternal smoking–related CpG sites that are differentially methylated with the same direction and of a similar magnitude from birth to childhood.^11,40^ Investigations of these specific CpG sites can be interesting, because these CpG sites may exert cumulative direct or indirect effects on the continuous brain development after birth. Furthermore, the substantially reduced sample size for the mediation analysis makes it difficult to capture subtle indirect effects.

### Limitations

Our study has several limitations. First, information on tobacco use was only collected with a questionnaire without using biomarkers such as plasma cotinine.^41^ In addition, using paternal smoking proceeding pregnancy to index paternal smoking during pregnancy might lead to misclassification, although evidence shows consistent smoking habits of the partner.^42^ Second, all of the children included in the DNA methylation sample in the Generation R Study were White; therefore, the results cannot be generalized to other racial or ethnic groups. Third, because this was an observational study, residual confounding may not be fully ruled out; thus, caution should be used when inferring causality. Future studies using complementary strategies (eg, sibling designs, mendelian randomization) to the paternal negative controls are warranted to strengthen causal inference. Additionally, the clinical implications of our findings are not explicit. Follow-up data collection on neurocognitive outcomes and the inclusion of functional brain measures such as electroencephalography or functional MRI are needed to unravel the clinical relevance of the observed differences in brain morphology.

## Conclusions

The findings of this study suggest that continued maternal tobacco use during pregnancy was associated with offspring brain development in preadolescence. Therefore, interventions targeting maternal smoking cessation before pregnancy or in early pregnancy may favor normal brain development among children in the long term.

## Notes

Supplement. **eMethods.** Parental Tobacco Use, Neuroimaging, and Statistical Analysis **eFigure 1.** Flowchart for Study Population Selection **eTable 1.** Nonresponse Analysis **eTable 2.** Association of Maternal Smoking During Pregnancy With Cortical Morphology in 10-Year-Old Children **eTable 3.** Demographic Information of Children With DNA Methylation (n = 784) **eFigure 2.** Mediation Analysis of Methylation Risk Score **eTable 4.** Association of Maternal Smoking During Pregnancy With Regional Brain Volumes in 10-Year-Old Children, Inverse Probability Weighted **eTable 5.** Association of Maternal Smoking During Pregnancy With Regional Brain Volumes in 10-Year-Old Children, Prenatally Included Only **eFigure 3.** Association of Maternal Smoking During Pregnancy With Cortical Morphology in 10-Year-Old Children, Prenatally Included Only
eReference
