# Supplementary material for: Association of Maternal Tobacco Use During Pregnancy With Preadolescent Brain Morphology Among Offspring
Source: JAMA Netw Open. 2022 Aug 1;5(8):e2224701. doi: 10.1001/jamanetworkopen.2022.24701 (PMC9344360; doi:10.1001/jamanetworkopen.2022.24701)
Supplement: Supplement. — eMethods. Parental Tobacco Use, Neuroimaging, and Statistical Analysis eFigure 1. Flowchart for Study Population Selection eTable 1. Nonresponse Analysis eTable 2. Association of Maternal Smoking During Pregnancy With Cortical Morphology in 10-Year-Old Children eTable 3. Demographic Information of Children With DNA Methylation (n = 784) eFigure 2. Mediation Analysis of Methylation Risk Score eTable 4. Association of Maternal Smoking During Pregnancy With Regional Brain Volumes in 10-Year-Old Children, Inverse Probability Weighted eTable 5. Association of Maternal Smoking During Pregnancy With Regional Brain Volumes in 10-Year-Old Children, Prenatally Included Only eFigure 3. Association of Maternal Smoking During Pregnancy With Cortical Morphology in 10-Year-Old Children, Prenatally Included Only eReference [file jamanetwopen-e2224701-s001.pdf]

Supplementary Online Content

Zou R, Boer OD, Felix JF, et al. Association of maternal tobacco use during pregnancy with preadolescent brain morphology among offspring. *JAMA Netw Open*. 2022;5(8):e2224701. doi:10.1001/jamanetworkopen.2022.24701

eMethods. Parental Tobacco Use, Neuroimaging, and Statistical Analysis
eFigure 1. Flowchart for Study Population Selection
eTable 1. Nonresponse Analysis
eTable 2. Association of Maternal Smoking During Pregnancy With Cortical Morphology in 10-Year-Old Children
eTable 3. Demographic Information of Children With DNA Methylation (n = 784)
eFigure 2. Mediation Analysis of Methylation Risk Score
eTable 4. Association of Maternal Smoking During Pregnancy With Regional Brain Volumes in 10-Year-Old Children, Inverse Probability Weighted
eTable 5. Association of Maternal Smoking During Pregnancy With Regional Brain Volumes in 10-Year-Old Children, Prenatally Included Only
eFigure 3. Association of Maternal Smoking During Pregnancy With Cortical Morphology in 10-Year-Old Children, Prenatally Included Only
eReference

This supplementary material has been provided by the authors to give readers additional information about their work.

© 2022 Zou R et al. *JAMA Network Open*.

eMethods. Parental Tobacco Use, Neuroimaging, and Statistical Analysis

Maternal and paternal tobacco use

Maternal tobacco use at enrollment was assessed in the first questionnaire by asking whether the mother smoked during pregnancy. In the second and third questionnaires (mid- and late pregnancy), mothers were asked whether they had smoked in the last 2 months. Mothers who reported no smoking or to have smoked until pregnancy was known in the first questionnaire, but acknowledged in the second or third questionnaire to have smoked during pregnancy were categorized to 'continued smoking during pregnancy'. When information was missing on maternal smoking at enrollment, information from the second and/or third questionnaire was used to classify the mothers into non-smokers or continued smokers. The frequency of tobacco use for the smoking mothers was categorized into 'less than 1 cigarette a day', '1-2 cigarettes a day', '3-4 cigarettes a day', '5-9 cigarettes a day', '10-19 cigarettes a day', and '20 or more cigarettes a day'.

For paternal tobacco use, the question for mothers was 'In the months preceding this pregnancy, did he (the biological father) smoke cigarettes, cigars or shag'; the question for participating partners was 'In the two months before the pregnancy of your partner, have you smoked'.

Neuroimaging

Parameters used for T_1-weighted image sequence: GE option BRAVO, TR = 8.77 ms, TE = 3.4 ms, TI = 600 ms, flip angle = 10°, matrix size = 220 × 220, field of view = 220 mm × 220 mm, slice thickness = 1 mm, number of slices = 230, ARC acceleration factor = 2.

Cortical reconstruction smoothing: thickness maps for each subject were smoothed with a 10 mm full-width half-maximum Gaussian kernel, and local gyrification index (LGI) maps were smoothed using a 5 mm full-width half-maximum Gaussian kernel.

Statistical analysis

Multiple comparison correction for surface-based analyses: clusterwise p-values were Bonferroni corrected for the two hemispheres ($p<0.025$), and a cluster forming threshold (CFT) of $p=0.001$ was selected for significance testing because it has shown high correspondence with actual permutation testing at the smoothing kernels used.[1]

© 2022 Zou R et al. *JAMA Network Open*.

eFigure 1. Flowchart for Study Population Selection

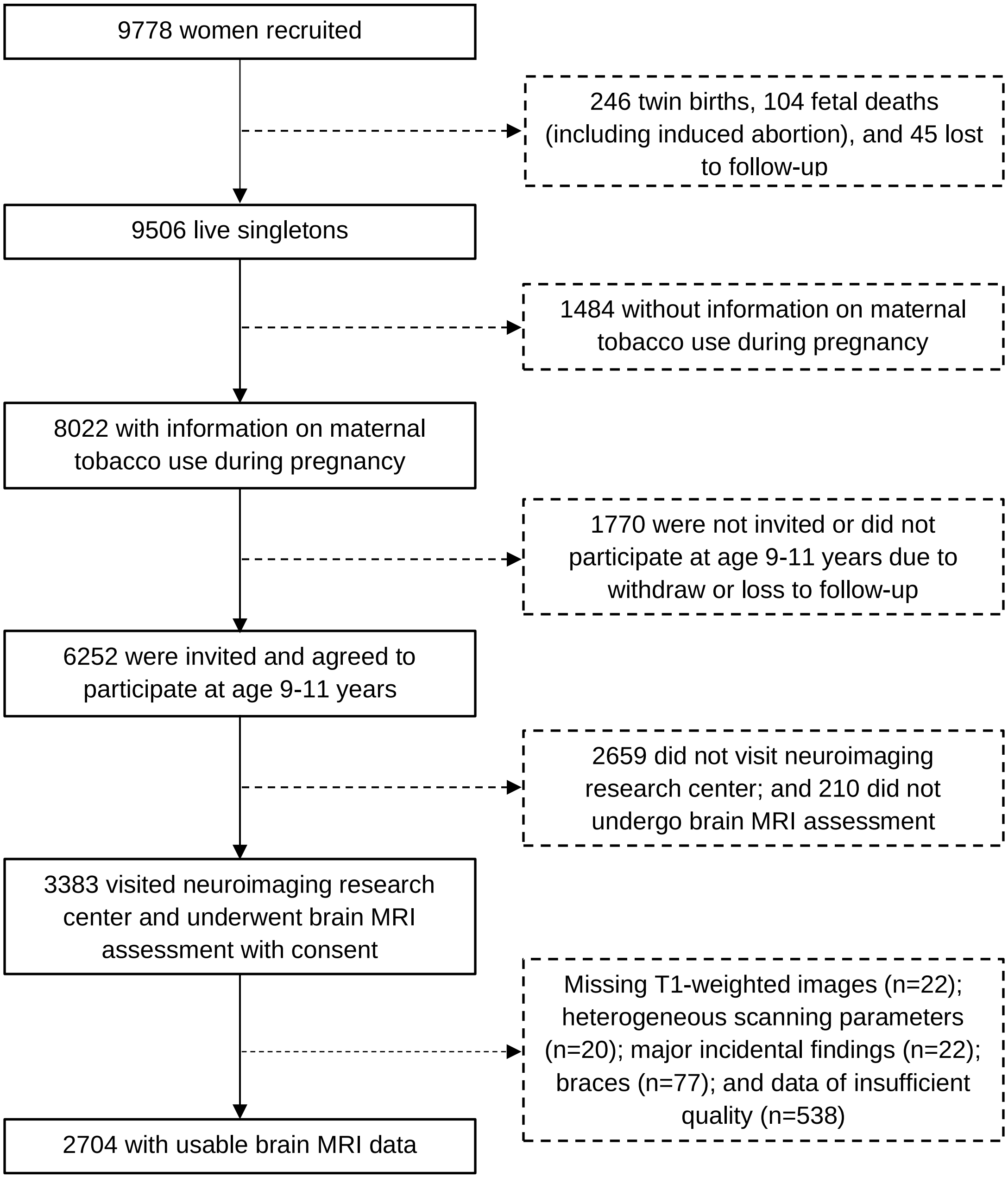

© 2022 Zou R et al. *JAMA Network Open*.

eTable 1. Nonresponse Analysis

**Characteristics**	**Respondents**[a] (n=2704)	**Non-respondents** (5318)	***P* value**[b]
**Maternal**			
Age at enrollment, mean (SD), years	31.1 (4.9)	29.3 (5.5)	<.001
Ethnicity, n (%)			
Dutch	1587 (58.7%)	2449 (46.5)	<.001
Non-Dutch Western	233 (8.6%)	464 (8.8)	
Non-Dutch non-Western	884 (32.7%)	2350 (44.6)	
Marital status (with partner), n (%)	2407 (89.0)	4355 (83.6)	<.001
Pre-pregnancy BMI, mean (SD)	23.4 (4.1)	23.7 (4.5)	.03
Parity (multipara), n (%)	1090 (40.3)	2374 (45.4)	<.001
Psychopathology score, mean (SD)[c]	0.3 (0.4)	0.3 (0.4)	<.001
Educational level, n (%)			
Primary or below	174 (6.4)	690 (13.5)	<.001
Secondary	1114 (41.2)	2521 (49.3)	
Higher	1416 (52.4)	1907 (37.3)	
Alcohol use during pregnancy, n (%)			
Never	1105 (40.9)	2623 (53.1)	<.001
Until pregnancy was known	392 (14.5)	639 (12.9)	
Occasionally	949 (35.1)	1383 (28.0)	
Frequently[d]	258 (9.5)	293 (5.9)	
Household net income, €/month, n (%)			
Less than 1200	420 (15.5)	896 (23.8)	<.001
1201-2000	435 (16.1)	765 (20.3)	
More than 2000	1849 (68.4)	2108 (55.9)	
Smoking during pregnancy, n (%)			
Never	2102 (77.7)	3793 (71.3)	<.001
Until pregnancy was known	238 (8.8)	442 (8.3)	
Continued	364 (13.5)	1083 (20.4)	
**Child**			
Sex (male), n (%)	1334 (49.3)	2718 (51.1)	.13

[a] Statistics of the first imputed dataset are reported. Percentages have been rounded and may not total 100.
[b] Continuous variables were compared using t-test or Wilcoxon test; categorical variables were compared using chi-square test.
[c] Scores range from 0 to 4, with higher scores indicating more clinically relevant psychological symptoms.
[d] Defined as ‘one or more glasses of alcohol per week in at least two trimesters’.

© 2022 Zou R et al. *JAMA Network Open*.

eTable 2. Association of Maternal Smoking During Pregnancy With Cortical Morphology in 10-Year-Old Children

Cortical metrics[a]	Brain region	Size (mm^2)	Coordinates			Mean coefficient	Clusterwise p-value
			X	Y	Z		
All children (n=2455)							
Thickness	Left inferior parietal	301.6	-32.0	-85.6	12.7	0.05	.0009
Surface area	Left inferior parietal	1081.9	-32.7	-77.5	39.9	-0.03	.0001
	Left middle temporal	987.1	-52.5	-32.8	-10.3	-0.02	.0001
	Left lateral occipital	313.8	-11.6	-96.5	-10.2	-0.03	.02
	Right inferior parietal	1674.3	43.2	-74.9	21.6	-0.04	.0001
	Right pericalcarine	1633.5	15.2	-92.5	-4.0	-0.04	.0001
Gyrification	Left postcentral	1017.2	-58.1	-9.4	16.8	-0.08	.0001
Children of prenatally included women (n=2268)							
Surface area	Left middle temporal	762.5	-53.6	-33.9	-10.2	-0.02	.0001
	Left inferior parietal	661.3	-39.7	-79.0	24.8	-0.03	.0003
	Right pericalcarine	1473.3	16.0	-92.8	-4.1	-0.04	.0001
	Right inferior parietal	1284.9	43.4	-75.5	21.5	-0.04	.0001
Gyrification	Left postcentral	789.3	-57.7	-8.9	16.5	-0.08	.0004

[a] Cortical metrics, including thickness, surface area and gyrification, in 10-year-old children born to women who continued smoking during pregnancy compared to those born to women who never smoked during pregnancy (2091 vs. 364 for analyses in all children, and 1918 vs. 350 for analyses in children of prenatally included women, respectively). Vertex-wise linear regression was used, and the presented model was adjusted for child sex and age at brain assessment and maternal ethnicity, age at enrollment, marital status, educational level, psychopathology score, alcohol use during pregnancy, and household income. All these clusters survived a clusterwise (Monte Carlo simulation with 5000 iterations) correction for multiple comparisons ($p < .001$). The corresponding brain regions are visualized in Figure 2.

© 2022 Zou R et al. *JAMA Network Open*.

eTable 3. Demographic Information of Children With DNA Methylation (n = 784)[a]

Characteristics	Descriptive statistics[b]
**Maternal**	
Age at enrollment, mean (SD), years	32.1 (4.0)
Marital status (with partner), n (%)	752 (95.9)
Pre-pregnancy BMI, mean (SD)	23.0 (3.8)
Parity (multipara), n (%)	294 (37.5)
Psychopathology score, mean (SD)[c]	0.2 (0.2)
Educational level, n (%)	
Primary or below	8 (1.0)
Secondary	241 (30.7)
Higher	535 (68.2)
Alcohol use during pregnancy, n (%)	
Never	200 (25.5)
Before pregnancy was known	113 (14.4)
Occasionally	357 (45.5)
Frequently[d]	114 (14.5)
Household net income, €/month, n (%)	
Less than 1200	16 (2)
1201-2000	76 (9.7)
More than 2000	692 (88.3)
Smoking during pregnancy, n (%)	
Never	617 (78.7)
Until pregnancy was known	78 (9.9)
Continued	89 (11.4)
**Child**	
Age at neuroimaging, mean (SD), years	10.2 (0.6)
Sex, number (%)	
Male	388 (49.5)
Female	396 (50.5)

[a] Only children of European ancestry were included.
[b] Statistics of the first imputed dataset are reported. Percentages have been rounded and may not total 100.
[c] Scores range from 0 to 4, with higher scores indicating more clinically relevant psychological symptoms.
[d] Defined as 'one or more glasses of alcohol per week in at least two trimesters'.

© 2022 Zou R et al. *JAMA Network Open*.

eFigure 2. Mediation Analysis of Methylation Risk Score

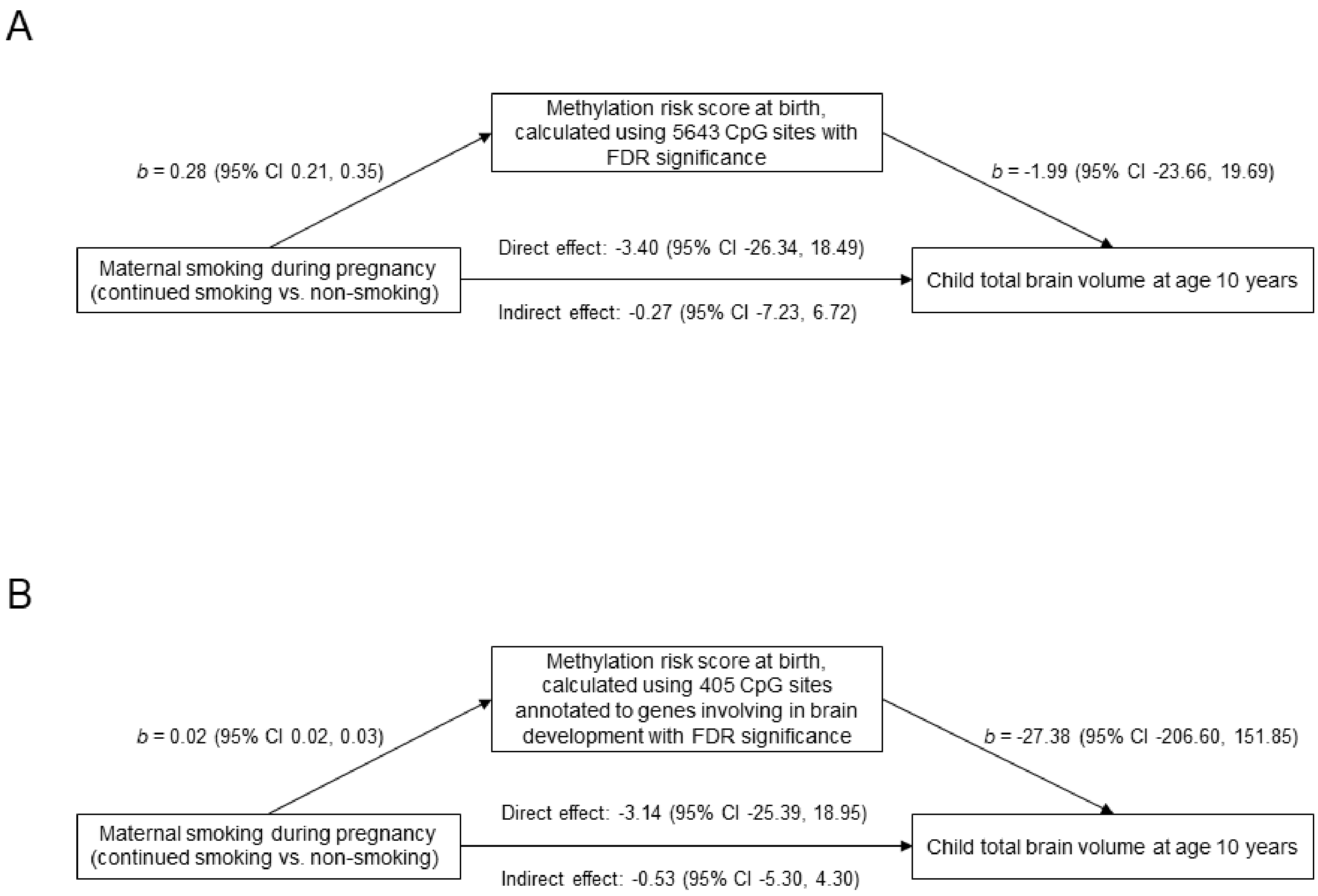

© 2022 Zou R et al. *JAMA Network Open*.

Legends: Based on findings from primary analyses, 681 (n=86 exposed to continued maternal smoking during pregnancy, and n=595 without exposure) of the 756 children of European ancestry with a methylation risk score and complete information on four principal genetic components were included in the analysis investigating whether DNA methylation risk score at birth mediates the association between maternal smoking during pregnancy and child brain volumes (here we used total brain volume as an example). Panel A illustrated results of methylation risk scores based on 5643 CpG sites that survived a false positive rate (FDR) correction; panel B illustrate results of methylation risk scores based on 405 CpG sites annotated to brain development pathway (http://amigo.geneontology.org/amigo/term/GO:0007420) that survived an FDR correction. Analyses were adjusted for child sex and age at neuroimaging and maternal age at enrollment, maternal educational level, cell types, plate number, and four genetic principal components.

© 2022 Zou R et al. *JAMA Network Open*.

eTable 4. Association of Maternal Smoking During Pregnancy With Regional Brain Volumes in 10-Year-Old Children, Inverse Probability Weighted[a]

**Maternal smoking during pregnancy**	**Cerebral gray matter volume**		**Cerebral white matter volume**		**Cerebellar volume**	
	b (95% CI)	p-value	*b* (95% CI)	p-value	*b* (95% CI)	p-value
Minimally adjusted model[b]						
Never	Reference	NA	Reference	NA	Reference	NA
Until pregnancy was known	1.4 (-5.2 to 7.9)	.68	-0.8 (-6.0 to 4.4)	.75	0.5 (-1.1 to 2.2)	.52
Continued	-15.6 (-21.2 to -10.0)	<.001	-10.7 (-15.4 to -6.1)	<.001	-2.4 (-3.8 to -1.0)	<.001
Fully adjusted model[c]						
Never	Reference	NA	Reference	NA	Reference	NA
Until pregnancy was known	0.4 (-6.0 to 6.7)	.91	-1.3 (-6.7 to 4.0)	.63	0.6 (-1.0 to 2.3)	.46
Continued	-8.4 (-14.0 to -2.8)	.003	-6.4 (-11.2 to -1.6)	.01	-0.9 (-2.3 to 0.5)	.23

Abbreviation: NA, not applicable.
[a] Linear regression was used. The *b* values represent volumetric differences (in cm^3) of the group that smoked until pregnancy was known (n=238) or the group that continued smoking (n=364) compared with the never smoked (reference) group (n=2102).
[b] Adjusted for child sex and age at brain assessment, and weighted by inverse probability to count for attrition.
[c] Adjusted for child sex and age at brain assessment and maternal ethnicity, age at enrollment, marital status, educational level, psychopathology score, alcohol use during pregnancy, household income, and weighted by inverse probability to count for attrition.

© 2022 Zou R et al. *JAMA Network Open*.

eTable 5. Association of Maternal Smoking During Pregnancy With Regional Brain Volumes in 10-Year-Old Children, Prenatally Included Only[a]

**Maternal smoking during pregnancy**	**Cerebral gray matter volume**		**Cerebral white matter volume**		**Cerebellar volume**	
	b (95% CI)	p-value	*b* (95% CI)	p-value	*b* (95% CI)	p-value
Minimally adjusted model[b]						
Never	Reference	NA	Reference	NA	Reference	NA
Until pregnancy was known	1.5 (-5.3 to 8.4)	.67	-1.4 (-7.2 to 4.5)	.65	0.7 (-1.0 to 2.3)	.42
Continued	-14.4 (-20.0 to -8.8)	<.001	-9.8 (-14.7 to -5.0)	<.001	-2.2 (-3.6 to -0.8)	.002
Fully adjusted model[c]						
Never	Reference	NA	Reference	NA	Reference	NA
Until pregnancy was known	0.2 (-6.5 to 6.8)	.96	-2.1 (-8.0 to 3.7)	.47	0.6 (-1.0 to 2.3)	.45
Continued	-7.4 (-13.1 to -1.7)	.01	-5.5 (-10.5 to -0.6)	.03	-0.8 (-2.2 to 0.6)	.24

Abbreviation: NA, not applicable.
[a] Linear regression was used. The *b* values represent volumetric differences (in cm^3) of the group that smoked until pregnancy was known (n=224) or the group that continued smoking (n=350) compared with the never smoked (reference) group (n=1929).
[b] Adjusted for child sex and age at brain assessment.
[c] Adjusted for child sex and age at brain assessment and maternal ethnicity, age at enrollment, marital status, educational level, psychopathology score, alcohol use during pregnancy, and household income.

© 2022 Zou R et al. *JAMA Network Open*.

eFigure 3. Association of Maternal Smoking During Pregnancy With Cortical Morphology in 10-Year-Old Children, Prenatally Included Only

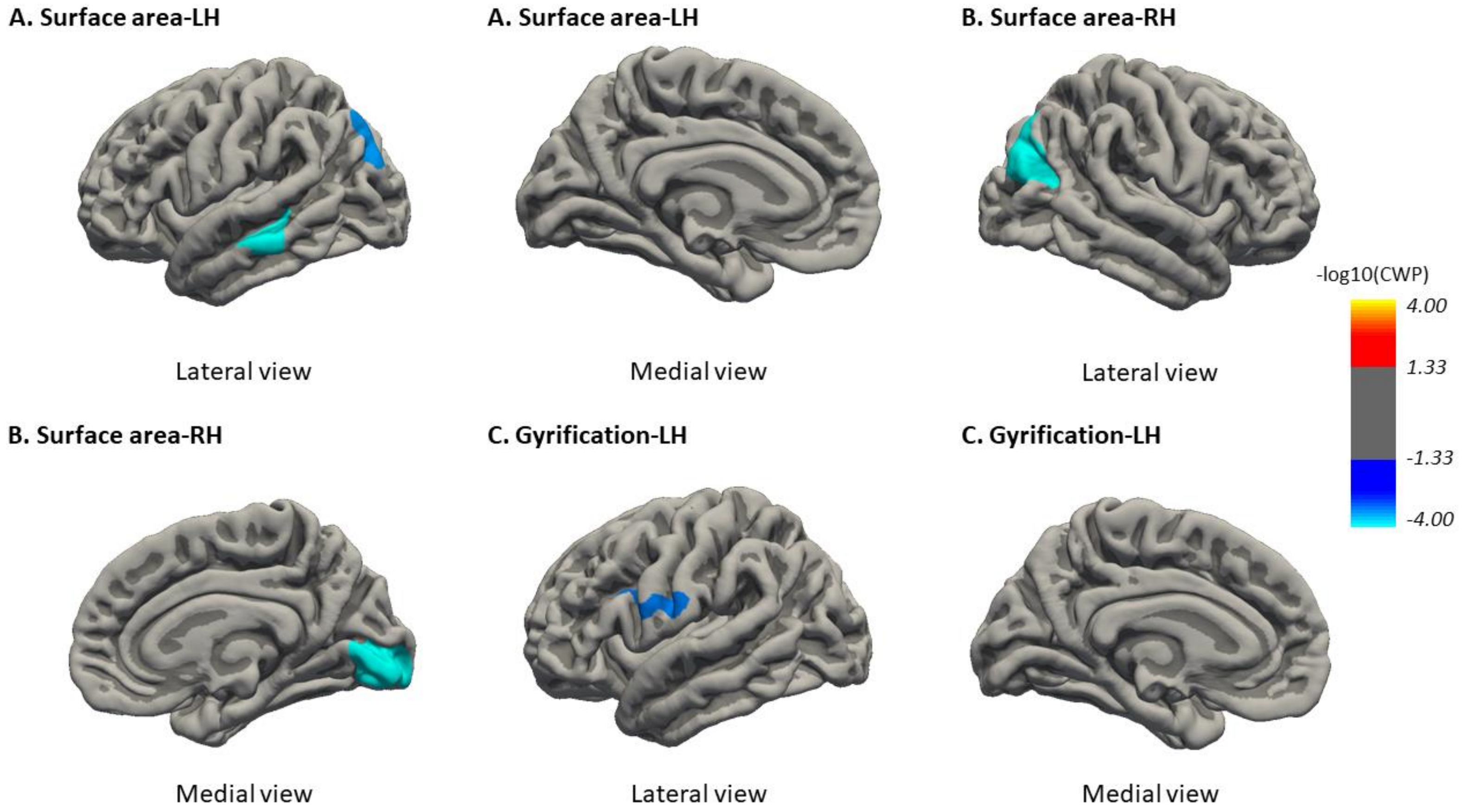

Legends: Cortical morphology (i.e., thickness, surface area, and gyrification) in 10-year-old children born to mothers who continued smoked during pregnancy (n=350) compared with those born to mothers who never smoked during pregnancy (i.e., reference, n=1918). Models were adjusted for child sex and age at brain assessment and maternal ethnicity, age at enrollment, marital status, educational level, psychopathology score, alcohol use during pregnancy, and household income. Clusters of red to yellow represent larger surface area or more gyrification; clusters of dark to light blue represent smaller surface area or less gyrification. The cortical differences in the colored clusters all survived a clusterwise (Monte Carlo simulation with 5000 iterations) correction for multiple comparisons ($p < .001$). CWP, clusterwise p-values; LH, left hemisphere; RH, right hemisphere.

© 2022 Zou R et al. *JAMA Network Open*.

eReference

1. Greve DN, Fischl B. False positive rates in surface-based anatomical analysis. *Neuroimage*. 2018;171:6-1

© 2022 Zou R et al. *JAMA Network Open*.
